# Planar array with bidirectional elements for tunnel environments

**DOI:** 10.1038/s41598-017-15817-4

**Published:** 2017-11-13

**Authors:** Ren Wang, Bing-Zhong Wang, Xiao Ding, Jun-Yu Ou

**Affiliations:** 1 Institute of Applied Physics, University of Electronic Science and Technology of China, Chengdu, 610054 China; 2 Optoelectronics Research Centre and Centre for Photonic Metamaterials, University of Southampton, Highfield, Southampton, SO17 1BJ UK

## Abstract

The growing demands for wireless communications in tunnel environments are driven by requests for maintaining uninterrupted internet access for users in tunnels as well as wireless connections for wireless sensors, security, and control networks. Nevertheless, wireless networks in tunnel environments are associated with technical challenges related to elongated wireless coverage in two opposite near-end-fire directions. Here, we introduce a low-profile bidirectional antenna that can be mounted on a large ground plane and that has a dual-magnetic-current mode exhibiting radiation patterns with 3-dB beamwidth coverage at near-end-fire angles. Furthermore, we realized a planar array with eight such bidirectional antennas that were configured as a sparse array in order to reduce the commonplace issues of mutual coupling and grating lobes. The radiation beams of the proposed antenna array can scan in the end-fire area (60° < φ < 120°, 45° < θ < 80°), with scanning gains of 11~15 dB in the near-end-fire directions. In addition, we demonstrate that the proposed array can adaptively generate a directional single beam or multiple beams according to the positions of users, which is suitable for intelligent communication systems with low power consumptions and high communication bandwidths in tunnel environments.

## Introduction

Tunnel environments, i.e., long and narrow path service areas, are common communication scenarios, and include subways, underground mines, streets, highways, corridors, and some special buildings^1–3^. In such environments, stretched wireless coverage in two opposite near-end-fire directions is highly needed. Therefore, directional antennas with bidirectional radiation patterns and high gains are a promising option. Combining two directional antennas or radiator arrays is a conventional method for generating bidirectional radiation patterns, and examples include combinations of two Yagi-type radiators^4,5^ and two patch radiators^6–8^. To increase radiation gain, two groups of cascaded radiator arrays can be combined^9–12^. Meander-line folded dipoles^9^, printed dipoles^10^, crossed dipoles^11^, and ring radiators^12^ have been cascaded for use as bidirectional antennas. In addition to combinations of two directional antennas, there are a few radiators that inherently emit bidirectional radiation. When used as a complementary structure of two opposite patches^13^, a metallic ring is a general structure for producing bidirectional beams. Probe excited rectangular or circular rings have been widely studied for many years^14–17^. In particular, ring antennas have the advantage of small cross-sections in the ventilation direction^18^ compared to a configuration of two opposite patches. Furthermore, advanced properties, such as wide operational bands^19^, dual-polarization^20^, and circular polarization^21^ can be obtained by modifying the feed probes of ring antennas. To optimize a ring antenna, a ring-turnstile-slot log-periodic antenna, which emits dual-circularly polarized bidirectional radiation, was recently proposed^22^. Slots on metallic sheets are another structure with bidirectional radiation patterns, examples of which include a bidirectional monopole-feeding slot antenna array^23^ and a coplanar waveguide slot array that were proposed to produce bidirectional radiation patterns^24^.

Although the above-mentioned antennas have superior bidirectional radiation performance in free space, they are less appropriate for on-wall or on-vehicle applications because they lack a large back ground plane to eliminate the influence of back structures and materials on radiation. As a result, a linear reconfigurable array with two isolated ports and four L-shaped monopoles vertical to a metallic ground was proposed; the array had two opposite end-fire radiation patterns that could be switched by switching the feeding signals at two ports^25^. However, the monopole elements have a high profile and cannot be easily fabricated using standard techniques compared to printed patch antennas. Printed leaky-wave antennas have low profiles, can be effortlessly-fabricated to generate near-end-fire radiation beams and can be backed by larger ground planes. Consequently, coplanar waveguide^26^ and substrate integrated waveguide^27^ approaches were proposed to design end-fire leaky-wave antennas. By using tapered substrate-integrated waveguides, leaky-wave antennas can also be designed for conformal applications^28^. A fixed-frequency beam-steering leaky-wave antenna was proposed that can scan its beams in near-end-fire directions using binary switches^29^. The combination of two groups of end-fire leaky-wave radiators can generate bidirectional radiation patterns. As a special kind of leaky-wave antenna, surface-wave antennas have recently been widely investigated to produce end-fire bidirectional radiation patterns^30–32^ using large ground planes^33^ or high impedance surfaces^34,35^. In addition, microstrip magnetic dipoles with a large ground plane are another method for generating near-end-fire radiation patterns with vertical polarizations^36–39^.

The above antennas and arrays that can be mounted on a large ground plane can produce near-end-fire radiation beams or bidirectional radiation beams, which are highly needed for tunnel environments. Nevertheless, it is much more energy-efficient to automatically direct radiation beams to users in modern intelligent communication systems. Therefore, phased arrays capable of beam steering are highly needed in communication systems^40–42^. When we focus on tunnel communication environments, a new challenge arises. Generally, phased arrays can scan their beams in broadside directions, and scanning beams in near-end-fire directions remains a technological challenge^43–45^. To solve that problem, some methods have been proposed to increase element beamwidth, including the high impedance surface method^46^, image-theory method^47^, magnetic current method^48^, and reconfigurable element method^49,50^ for open space applications. In addition to approaches for widening element beamwidths, metallic wall^51–56^ and wide-angle impedance matching (WAIM) approaches^57–59^ were proposed to match the active impedances of elements at different scanning angles. However, those widening element beamwidth approaches are not adapted to tunnel environments because scanning range in broadside directions is redundant in tunnel environments and the arrays can only scan beams in a certain plane rather than over bidirectional areas.

In this paper, a low-profile, easily fabricated dual-mode bidirectional antenna and a planar phased array with bidirectional elements are proposed for tunnel environments. The design methodology is first analysed considering three important key factors that are introduced to guide the design of an array for use in a tunnel: designing bidirectional elements, dealing with the strong coupling, and avoiding grating lobes. Furthermore, a dual-magnetic-current antenna with two bidirectional modes is proposed based on the operational principle of phase-inversed binary arrays. By using a patch with opposite short edges and opposite open edges, a dual-magnetic-current antenna is realized. The 3-dB beam-width can cover bidirectional near-end-fire directions from 40° to 85° and from −40° to −85°. A planar array with eight bidirectional elements is then presented. Decoupling cavities and a sparse array configuration are used to achieve weak mutual coupling and avoid grating lobes in the array. Finally, the time reversal synthesis method is applied to adaptively synthesize single beam and multiple beams according to the users, which is suitable for intelligent communication systems used in tunnel environments.

## Design Procedures and Results

### Design procedures

**Figure 1:**
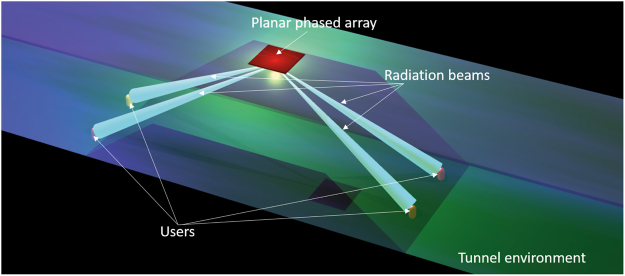
Schematic diagram of a planar phased array used in tunnel environment. Array is installed on the top of tunnel and its radiation beams should direct to near-end-fire directions. Ideally, radiation beams of the tunnel-used array should be automatically directed to users.

The arrays used for tunnel environments in modern intelligent communications systems should have three key performance traits: (1) large back ground planes for on-wall or on-vehicle applications, (2) the ability to generate radiation that can cover near-end-fire directions, (3) ideally, the radiation beams that are automatically directed to users automatically. The schematic diagram of the planar phased array for use in tunnel environments is shown in Fig. 1.

To design an array for use in tunnels, there are three challenges: (1) designing low-profile bidirectional elements with a large back ground plane, (2) dealing with the strong coupling between bidirectional elements, especially in the along-tunnel direction and (3) avoiding grating lobes and large sidelobes. To homogenize the radiation distribution in a tunnel, the radiation pattern of each antenna array should cover its near-end-fire directions, and the directions along the broadside of one antenna array are covered by another antenna array, which means that the broadside radiation patterns of the array elements are redundant and that the bidirectional element is suitable for tunnel applications. To design the bidirectional antennas with large ground planes, a dual-magnetic-current structure based on an inversed-phase binary array is proposed in this paper. The radiation pattern of the bidirectional element is mainly focused in the two near-end-fire directions along the tunnel; therefore, the coupling between the elements arranged along the tunnel direction may be very strong. In this paper, two techniques are used to decrease coupling between elements. First, a grounded decoupling cavity is placed around the elements, and second, the distance between the elements along the tunnel direction is approximately one wavelength. However, according to the product principle of array patterns, grating lobes may appear when the distance between array elements exceeds a half wavelength. Therefore, decreasing coupling is contradictory to avoiding grating lobes. To solve these problems, we propose that the elements along the vertical direction to the tunnel are arranged as stair steps, such that the element distance in the tunnel direction decreases and grating lobes can be avoided.

### Dual-mode dual-magnetic-current bidirectional antenna

**Figure 2:**
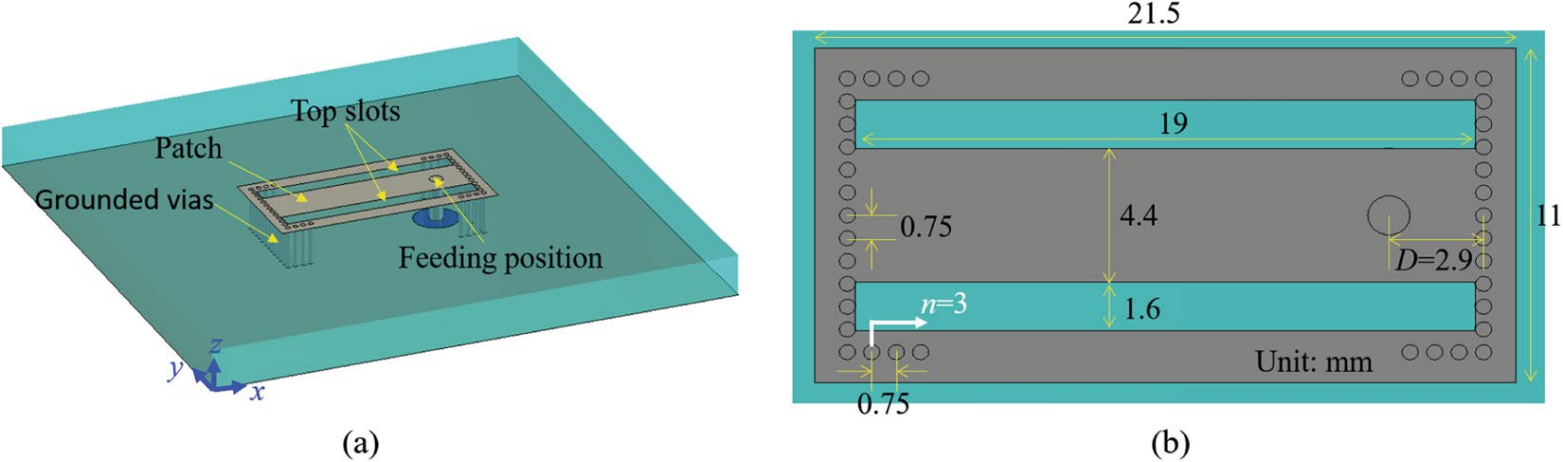
Geometry of the dual-mode bidirectional antenna. (**a**) Perspective view and (**b**) partial enlarged detail of the antenna. The diameter of the grounded vias is 0.5 mm. The color gray represents copper material and the color aquamarine represents dielectric substrate.

The geometry of the bidirectional antenna is shown in Fig. 2, and its optimum dimensions are indicated in the figure. A patch with two slots is printed on a substrate with a thickness of 4 mm and a relative permittivity of 2.65. Two edges of the patch are shorted and the other two edges are partially shorted at the ends. The open area of two opposite edges can support two inversed equivalent magnetic currents, which compose an inversed-phase binary array and produce a bidirectional radiation pattern. In this antenna, there are two close resonant modes, which are denoted TM12 and TM14. The two slots on the patch are used to excite the TM14 mode, which has a resonance frequency close to that of TM12. The feed position is at the centre of the patch in the y direction but not at the centre in the x direction. The operational principle will be analysed step by step below.

**Figure 3:**
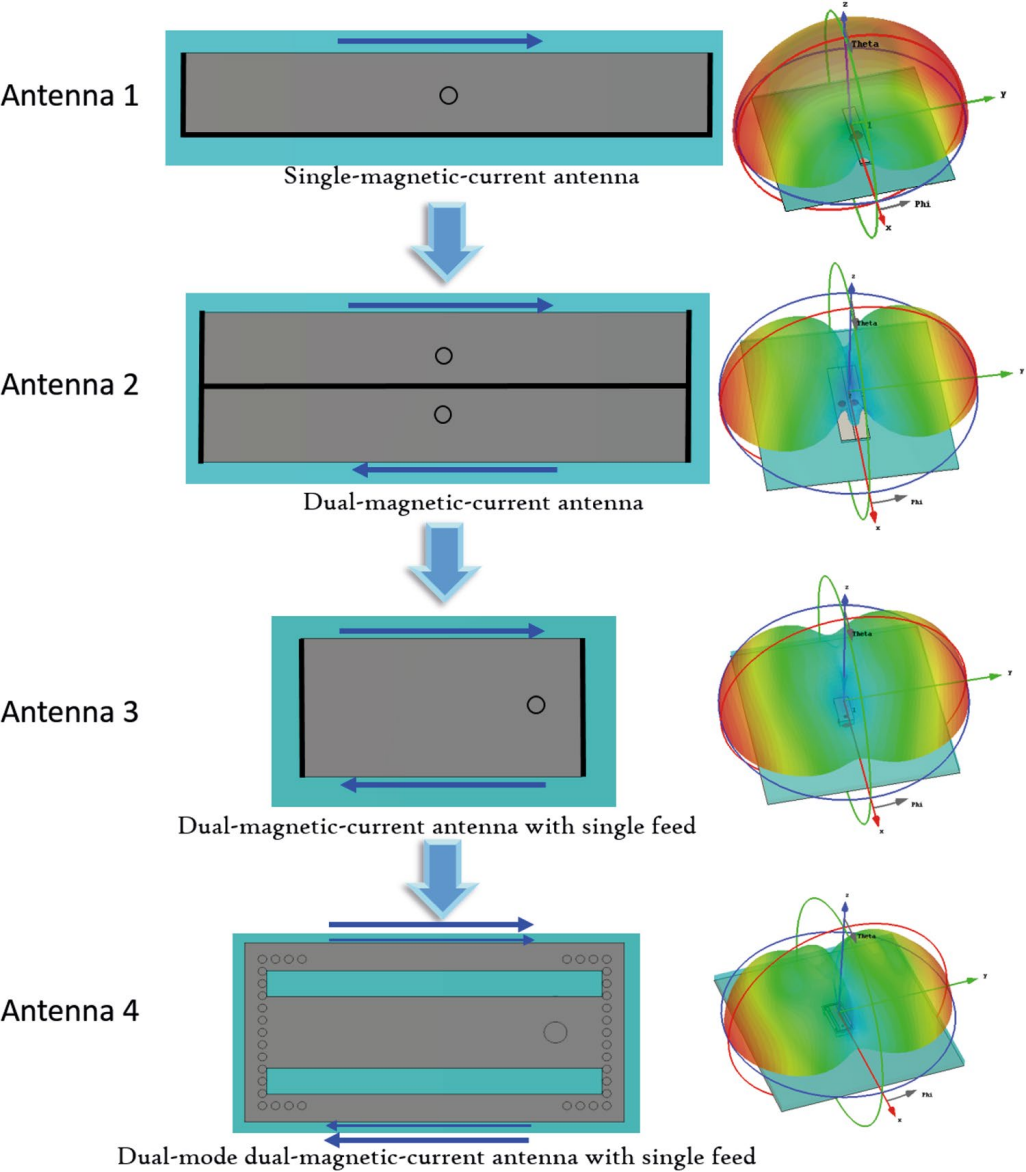
Evolution of magnetic-current antennas. The left column, middle column, and the right column are the names, structure diagrams, and corresponding radiation patterns, respectively. The blue arrows indicate the equivalent magnetic currents. In the simulations, the ground is set to infinite to clearly show performances of radiation patterns and avoid the influence of ground edges. Antenna 1, i.e., single-magnetic-current antenna, has an open edge and three short edges. The slot on open edge can be equivalent to a magnetic current and generate a dipole-like radiation pattern. Antenna 2 is composed of two single-magnetic-current antennas, which are fed with equal magnitude and phase. Two inversed equivalent magnetic currents are generated on the two open edges and the binary magnetic-current array has a bidirectional radiation pattern. Antenna 3 with two open edges and two short edges is a simplified structure of Antenna 2. Antenna 3 has only one feed and the impedance can be matched by adjusting the feed position. Antenna 4 is a practical antenna proposed in this paper. The sheets on short edges of Antenna 3 are replaced with metal grounded holes and two slots are etched on top patch to excite two binary magnetic-current modes and increase the operational frequency band.

To clearly display the operational principle of the proposed bidirectional antenna, the evolutionary process of magnetic-current antennas is shown in Fig. 3. Antenna 1 is a fundamental magnetic-current antenna and consists of an open edge and three short edges. The open edge can be equivalent to a magnetic current and generate a dipole-like radiation pattern. The fields on the open edge of the patch can be equivalent to a magnetic current by analogy to the radiation principle of slot antennas:

1 $${J}_{m}=-\,{n}_{y}\times {E}_{t}$$

where, ${n}_{y}$ is a unit vector in the z direction and ${E}_{t}$ is the electric field in the slots. According to the operational principle of inversed-phase binary arrays, two single-magnetic-current antennas are arranged back-to-back and are labeled Antenna 2 in the figure. When the two single-magnetic-current antennas of Antenna 2 are fed with equal magnitude and phase, the equivalent magnetic currents on the two open edges will have inversed phase because of the opposite directions of the open edges. As we know, inversed-phase binary array can produce a bidirectional radiation pattern, and Antenna 2 therefore emits a bidirectional radiation pattern. The structure of Antenna 3, which has two open edges and two short edges, is a simplification of the structure of Antenna 2. Antenna 3 has only one off-centre feed, the position of which controls impedance matching. Antenna 4 is an example of the practical antenna shown in Fig. 2. The sheets on the short edges of Antenna 3 are replaced with grounded holes, and the two slots that are etched on the top patch excite two binary magnetic-current modes and widen the operational frequency band.

**Figure 4:**
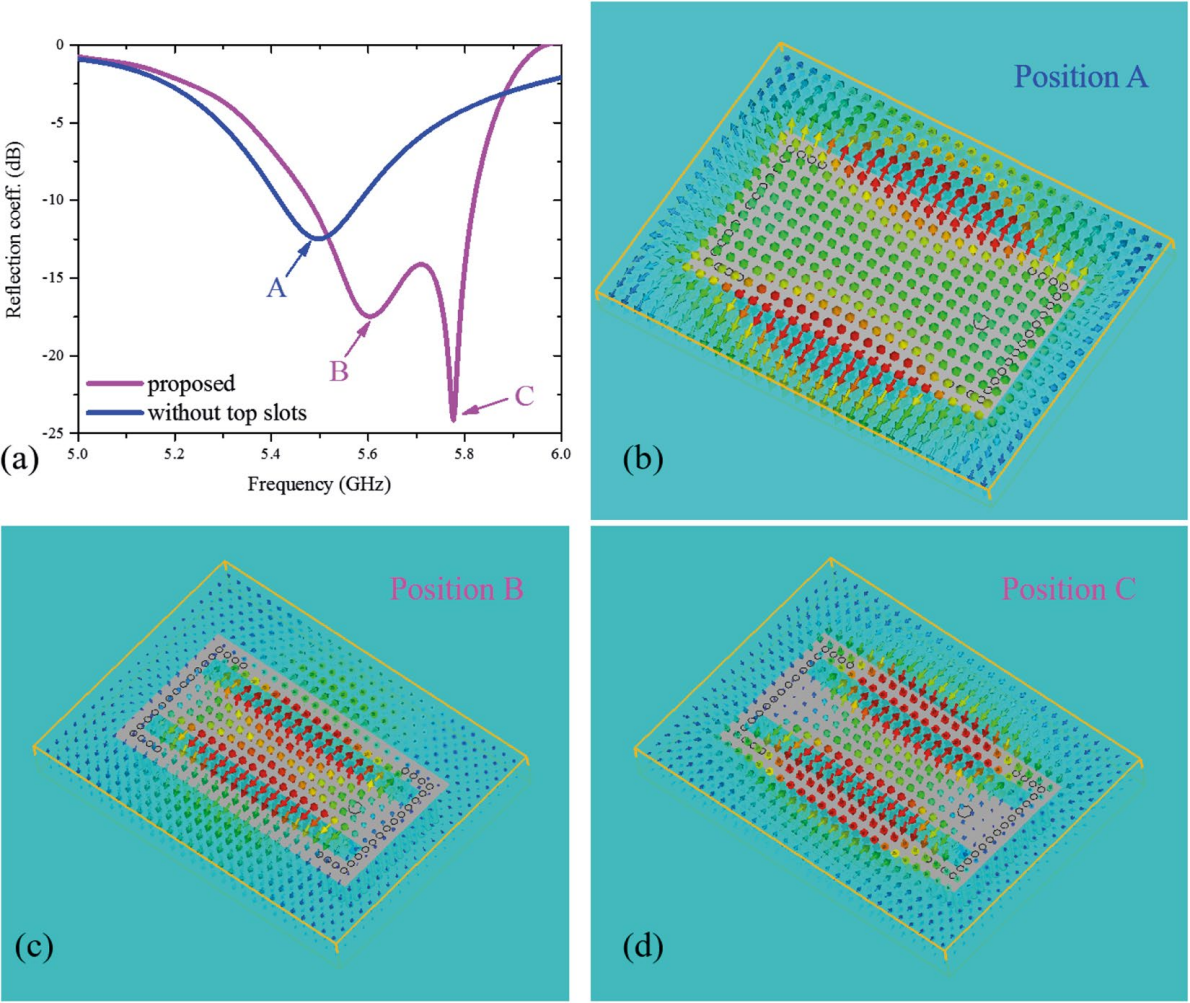
Operational principle of the dual-mode bidirectional antenna. (**a**) Reflection coefficients of the antenna shown in Fig. 3 with and without top slots. When there are two slots on the top patch, as shown in Fig. 3, two resonant points can be seen. When there is no top slot, only one resonant point can be seen. Electric field distributions at Positions A, B, and C are shown in (**b**), (**c**), and (**d**), respectively. The modes at Positions A and B are TM12 mode, and the mode at Position C is TM14 mode.

The reflection coefficients and electric field distributions of the bidirectional antenna in Fig. 2 with/without slots on the patch are shown in Fig. 4. When there are two slots on the top patch, which is the structure shown in Fig. 2, we can see two resonant valleys at 5.61 GHz (Position B) and 5.78 GHz (Position C), and when there are no slots on the patch, only one resonant valley at 5.48 GHz (Position A) can be seen. At Position A, the electric field distributions are from the ground to the patch in the central area and from the patch to the ground at the open edges, which indicates a TM12 mode. At Position B, the electric field distributions are similar to those at Position A. At Position C, the electric field distributions in the central area and at the open edges are from the ground to the patch, and the electric fields between the open edges and slots are from the patch to the ground, which indicates a TM14 mode. Therefore, the modes at Positions A, B, and C are the TM12 mode, the TM12 mode, and the TM14 mode, respectively. The slots on the patch result in a higher-order mode close to the resonant frequency of TM12 mode, and the two resonant modes induce a relatively wide operational frequency band.

**Figure 5:**
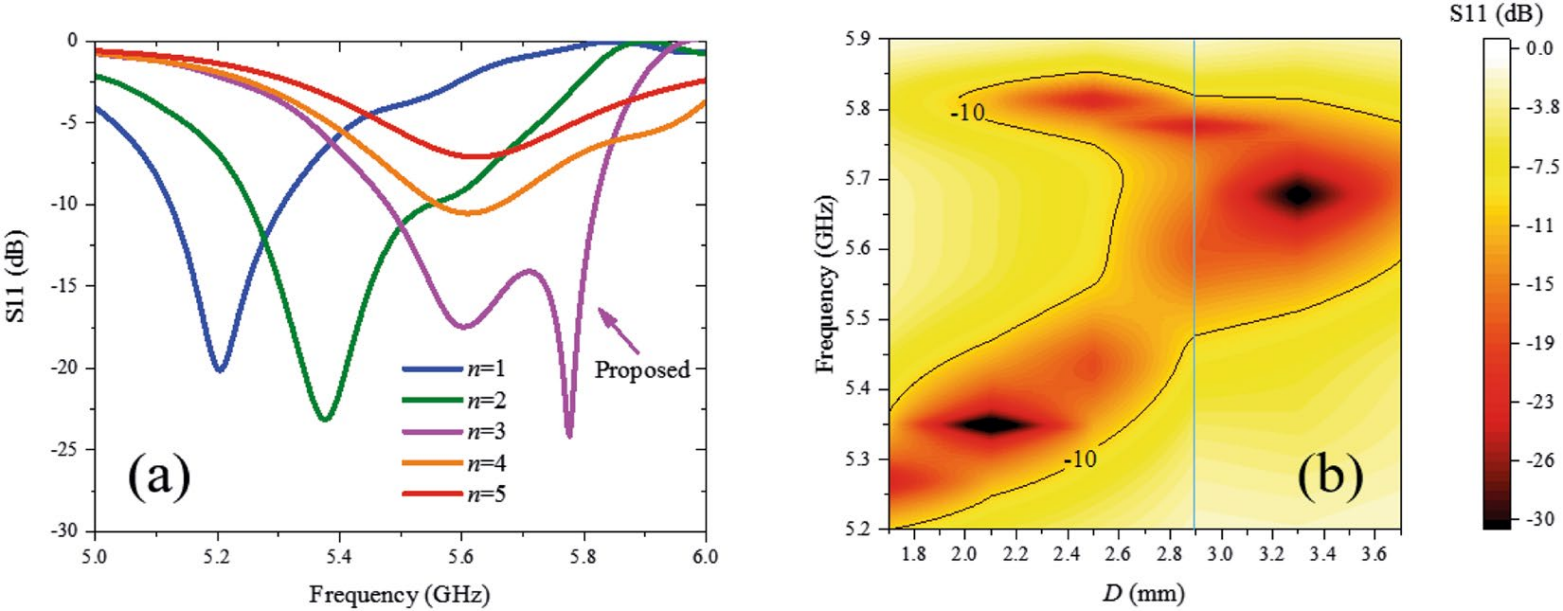
Relations between structure parameters and reflection coefficients. (**a**) Reflection coefficient versus the number *n* of side additional grounded holes. When *n* increases from 1 to 3, the resonant point of TM14 mode appears gradually; when *n* further increases from 3 to 5, the resonant depth decreases gradually. (**b**) Reflection coefficients versus different feed positions. With increasing distance *D* between feed position and grounded holes, two resonant points in the map gradually get closer. When *D* = 2.9 mm, a relatively wide operational frequency band is obtained, as the vertical line shows.

Aided by the slots on the top patch, the two modes resonate at two frequencies that are close to each other. To increase the operational frequency bandwidth, the feed position and grounded holes at the ends of the open edges are also important for impedance matching. From Fig. 2, we can see that there are 3 grounded holes (noted as side additional grounded holes) at each end of the two open edges, in addition to the grounded holes at the two short edges on the patch. The relation between the reflection coefficient and the number of additional side grounded holes, *n*, is shown in Fig. 5(a). When *n* increases from 1 to 3, the resonant depth of the TM14 mode gradually increases, and when *n* further increases from 3 to 5, the resonant depths of the two modes gradually decrease. The relation between the reflection coefficient and feed position is shown in Fig. 5(b). With increasing distance *D*, the two resonant points in the map gradually become closer. For *D* ~ 2 mm, the antenna is matched to the TM12 mode, for *D* ~ 3.3 mm, the antenna is matched to the TM14 mode, and for intermediate values of *D*, the antenna has a compromised impedance matching between the two resonant modes. When *D* = 2.9 mm, a relatively wide operational frequency bandwidth can be achieved, as the vertical line shows.

**Figure 6:**
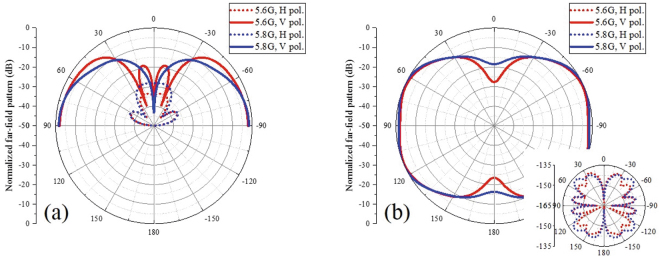
Simulated normalized patterns of the dual-mode bidirectional antenna with an infinite ground: (**a**) in the yz plane and (**b**) xy plane. For this antenna, the yz plane and xy plane are the E plane and H plane, respectively. The H pol. and V pol. in the figure represent the horizontally polarized component and vertically polarized component, respectively. The radiation at both 5.6 GHz and 5.8 GHz are bidirectional and the horizontally polarized component is much smaller than the vertically polarized component.

The simulated normalized patterns of the proposed dual-mode bidirectional antenna with infinite ground plane are shown in Fig. 6. For this antenna, the yz and xy planes are the E and H planes, respectively. In the yz plane, the vertically polarized components are shaped like the letter “m” at both 5.6 GHz and 5.8 GHz. The horizontally polarized component at 5.6 GHz is less than −30 dB, and that at 5.8 GHz is less than −25 dB. In the xy plane, the vertically polarized components are shaped like the number “8” at both 5.6 GHz and 5.8 GHz, and the horizontally polarized components are very small around the two end-fire directions. Figure 6 indicates that the radiation is bidirectional over a relatively wide frequency range, and the horizontally polarized component in the near-end-fire directions is much smaller than the vertically polarized component.

**Figure 7:**
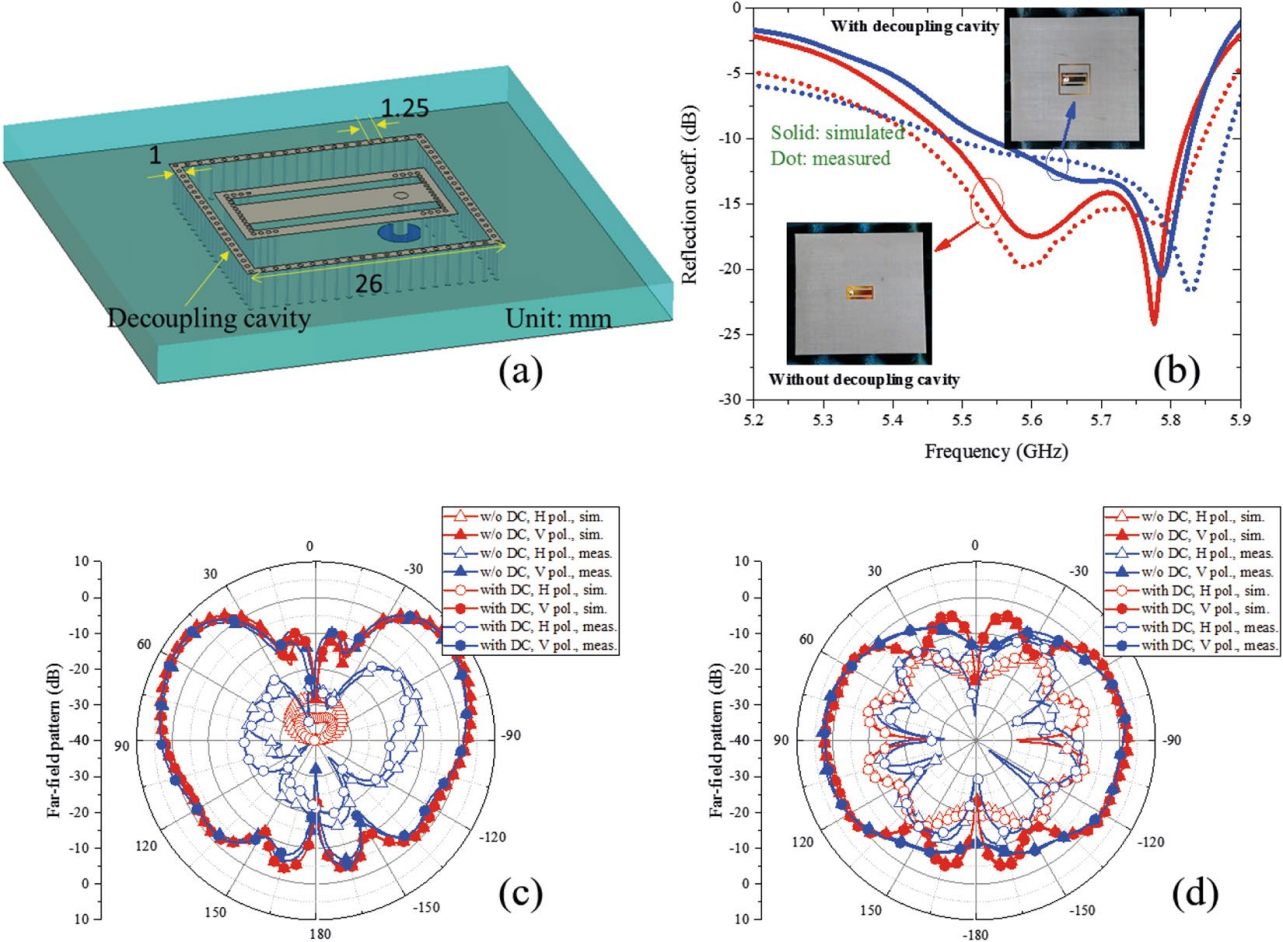
Performances of the dual-mode bidirectional antenna. (**a**) Geometry of bidirectional antenna with a decoupling cavity. (**b**) Simulated and measured reflection coefficients. The embedded figures are photographs of the bidirectional antennas with and without decoupling cavity. Both the two fabricated antennas have a 104 × 104 mm^2^ metallic ground. The simulated and measured operational frequency bands with S11 below −10 dB of the antenna with decoupling cavity are respectively 5.55–5.83 GHz and 5.5–5.88 GHz, which are a little smaller than that of the antenna without decoupling cavity. (**c**) and (**d**) are far-field radiation patterns at 5.8 GHz in the yz plane and xy plane, respectively. The DC, H pol., V pol., sim., and meas. in the figure represent the decoupling cavity, horizontally polarized component, vertically polarized component, simulated, and measured, respectively. The decoupling cavity has little effect on radiation patterns.

For array applications, a decoupling cavity is placed around the radiation patch of the proposed antenna to decrease the coupling between elements in the array, as shown in Fig. 7. The decoupling cavity is composed of a square strip and grounding holes. Except for the decoupling cavity, the rest of the structure and its dimensions are the same as in Fig. 2. Prototypes of the proposed dual-mode dual-magnetic-current bidirectional antenna with and without decoupling cavity were fabricated using standard printed circuit board techniques and the simulated and measured reflection coefficients are shown in Fig. 7(b). The simulated and measured operation frequency bands with S11 below −10 dB of the antenna with a decoupling cavity are 5.55–5.83 GHz and 5.5–5.88 GHz, respectively, and the simulated and measured bands with S11 below −10 dB of the antenna without a decoupling cavity are 5.5–5.82 GHz and 5.45–5.85 GHz, respectively. Compared to the case without the decoupling cavity, the first resonant depth of the antenna with decoupling cavity is deviated a small amount because of the effect of the decoupling cavity. The measured and simulated results agree well, with small errors resulting from the machining and measurements. It should be noted that the impedance matching can be further improved through additional tuning of the feed point and the side vias. In our design process, after adding the decoupling cavity to the antenna, we decide whether to adjust the feed point and the side vias depending on the condition of impedance matching degradation. If the operational frequency bandwidth changes by a large amount, we adjust the parameters to re-realize a good impedance matching, but if the operational frequency bandwidth changes little, we do not adjust the parameters. In the design example, although the decoupling cavity leads to degradation in impedance matching around the TM12 resonance, the entire operating bandwidth of the antenna changes little, and the degradation is acceptable; therefore, the structure parameter is not re-adjusted after adding the decoupling cavity. The far-field radiation patterns at 5.8 GHz in the yz and xy planes are shown in Fig. 7(c) and (d), respectively. The main polarized components– the vertically polarized components– of the antennas with and without the decoupling cavity almost overlap in both the yz plane and the xy plane, which indicates that the decoupling cavity has little effect on the radiation patterns. In the yz plane, compared to the infinite ground case shown in Fig. 6, the maximum radiation directions change from ±90° to ±55° because of the effect of the ground plane edges. The 3-dB beam-width of the vertically polarized component can cover bidirectional near-end-fire directions, from 40° to 85° and from −40° to −85°. The measured horizontally polarized components in both cases (with and without the decoupling cavity) are 15 dB smaller than the vertically polarized components. The radiation gain of the antennas can reach 7.5 dB. In the xy plane, the vertically polarized components are approximately 10 dB larger than the horizontally polarized components around the two end-fire directions, from 60° to 120° and from −120° to −60°. An excellent bidirectional radiation pattern is realized by using the proposed dual-magnetic-current structure.

From the above analysis, we can see that the significant challenge in designing low-profile bidirectional elements for use in tunnel arrays can be overcome by using the proposed dual-magnetic-current method. In the next section, a planar array with bidirectional elements will be proposed for use in tunnel.

### Array with bidirectional elements for use in tunnels

**Figure 8:**
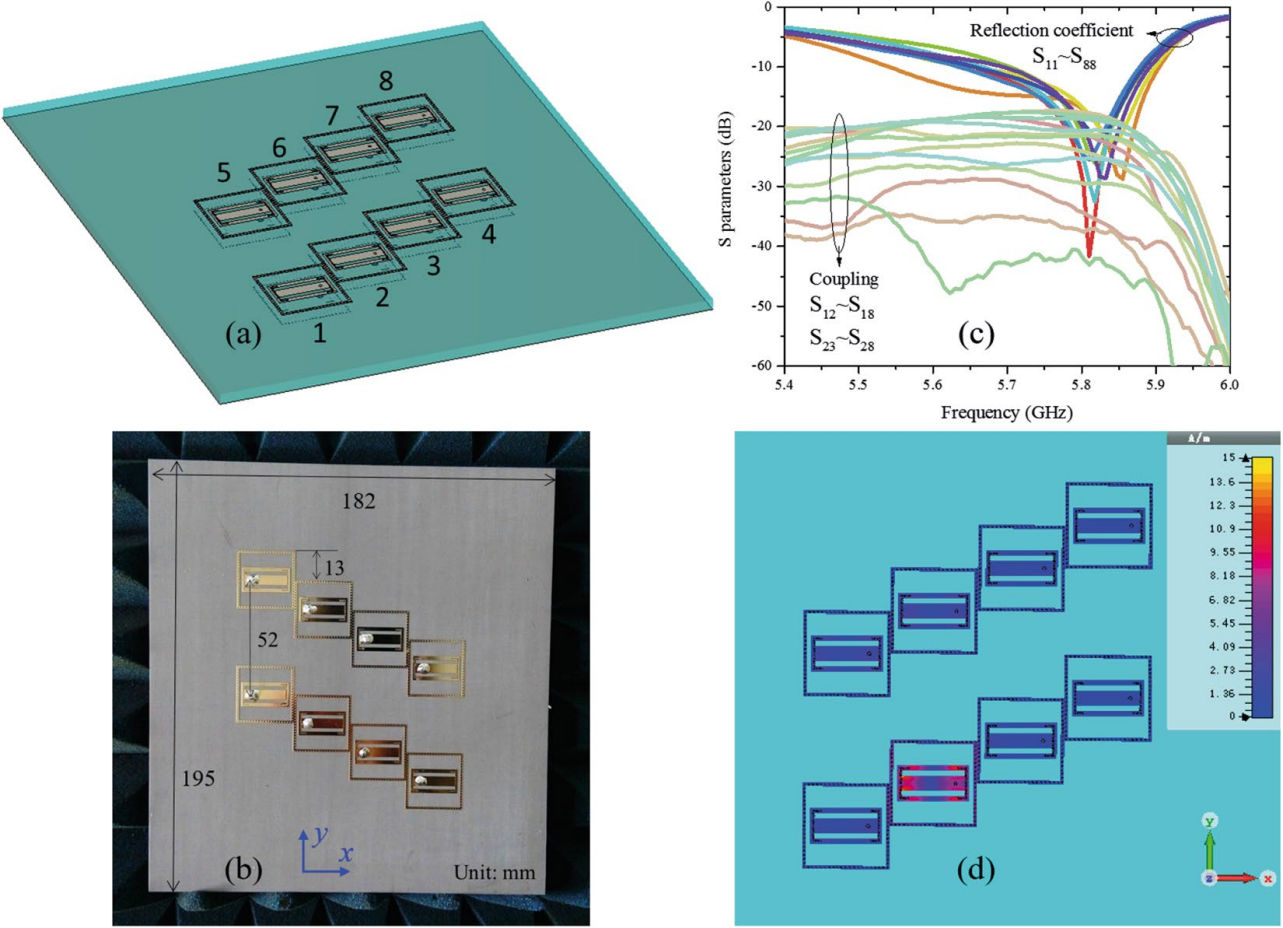
Planar sparse array with the bidirectional elements. (**a**) Geometry and (**b**) photograph of the proposed planar array. In the y direction, the distance between elements is approximately a wavelength at 5.8 GHz to decrease mutual coupling. In the x direction, elements are not arranged in a line to decrease mutual coupling and avoid grating lobes. (**c**) Some measured active S parameters. All the reflection coefficients are smaller than -10 dB over 5.75–5.86 GHz and the couplings between elements are smaller than −15 dB. (**d**) Magnetic field distribution plots at 5.8 GHz when Element 2 is excited and the other elements are terminated in matched loads.

The eight elements shown in Fig. 7(a) were arranged as a planar array, as shown in Fig. 8. From the last section, we know that two main beams of the bidirectional elements are located in the yz plane and are directed close to ±y directions. Therefore, the coupling between elements arranged along the y axis may be very strong, even with a decoupling cavity. To decrease the coupling, the element distance is set to 52 mm, which is approximately one wavelength at 5.8 GHz. However, on the basis of the product principle of array patterns, grating lobes may appear when the distance between array elements exceeds a half wavelength. To solve this problem, the elements along the x direction are arranged as stair-steps. Therefore, the element distance in the y direction is smaller than half wavelength. This sparse distribution allows the grating lobes to be avoided and makes the coupling between the elements in x the direction to decrease. The details of the array arrangement will be analysed below. The measured active S parameters of the array are shown in Fig. 8(c). All the reflection coefficients are smaller than −10 dB from 5.75–5.86 GHz, and the couplings between elements are smaller than −15 dB. The weak coupling between the elements indicates that the decoupling cavity and sparse arrangement are effective, which can also be verified by the magnetic field distribution plots shown in Fig. 8(d).

**Figure 9:**
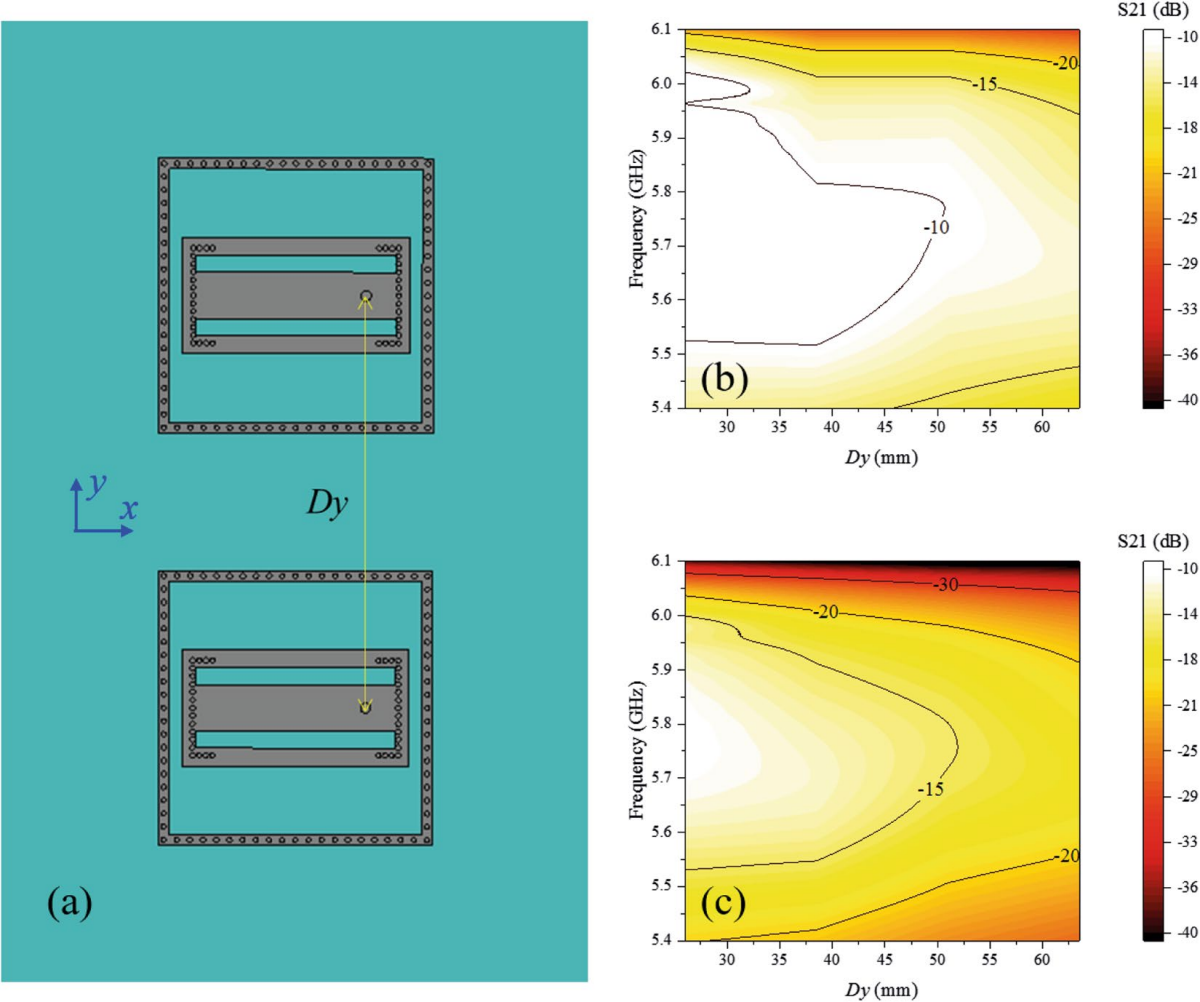
Relations between the distance *Dy* and element coupling. (**a**) Schematic of the array with two elements arranged along the y direction. (**b**) *S21* versus different distance *Dy* when the antennas have no decoupling cavities. (**c**) *S21* versus different distance *Dy* when the antennas have decoupling cavities. The mutual coupling decreases with the distance *Dy* in both two cases, and *S21* map in (**c**) is approximately 5 dB weaker than that in (**b**), which means the decoupling cavity can weaken the mutual coupling.

**Figure 10:**
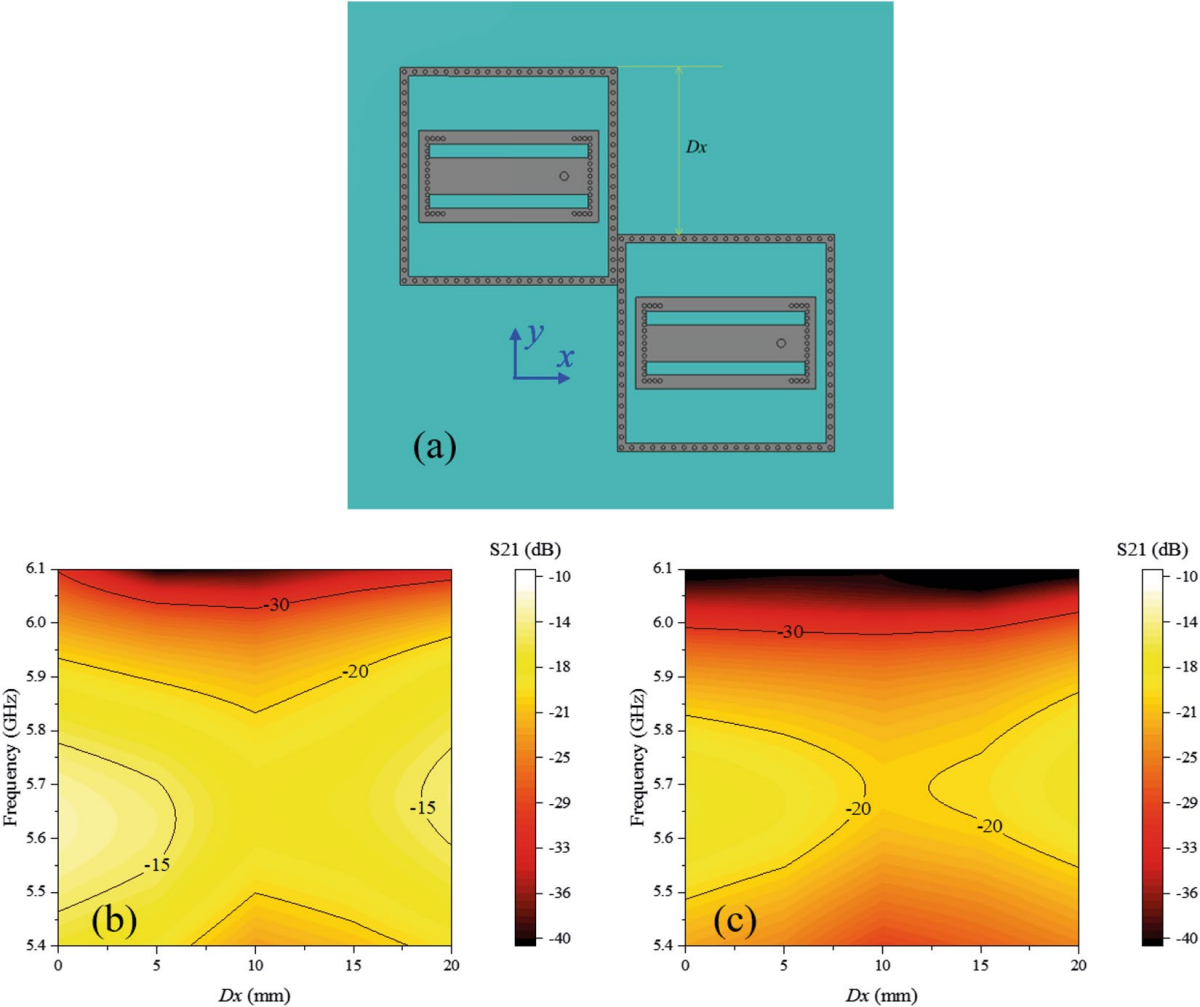
Relations between the distance *Dx* and element coupling. (**a**) Schematic of the array with two end-to-end elements. (**b**) *S21* versus different distance *Dx* when the antennas have no decoupling cavities. (**c**) *S21* versus different distance *Dx* when the antennas have decoupling cavities. In the operational frequency band, the mutual coupling decreases when the distance *Dx* increases from 0 to 10 mm and then increases. *S21* map in (**c**) is approximately 3 dB weaker than that in (**b**), which means the decoupling cavity can weaken the mutual coupling.

To clearly show the effect of the decoupling cavity and element arrangement on array coupling, two elements arranged side by side and two elements arranged end to end were analysed, as shown in Figs 9 and 10, respectively. From Fig. 9, we can observe that the mutual coupling decreases with distance *Dy* in both two cases, with and without the decoupling cavities, and S21 in the case with the decoupling cavity is approximately 5 dB weaker than that in the case without the decoupling cavity, which means that both the decoupling cavity and the large element distance can weaken mutual coupling. From Fig. 10, we can see that S21 in the case with the decoupling cavity is approximately 3 dB weaker than that in the case without the decoupling cavity, which is similar to Fig. 9. However, the trend in the variation in mutual coupling with *Dx* differs from that with *Dy*. In the operational frequency band, the mutual coupling decreases when the distance *Dx* changes from 0 to 10 mm and then increases in both two cases (with and without decoupling cavities). When *Dx* is near 0, the distance between the two elements is very small, and the coupling is therefore strong; as *Dx* increases, the distance increases, and the coupling decreases. However, when the right element moves further in the –y direction, the direction of the radiation emitted by the proposed bidirectional element becomes significant. We know that the radiation of the element is near the ± y direction. In the near field, the electric fields are strong around the open edges and weak around the short edges, as shown in Fig. 4. When *Dx* increases above 10 mm, the two elements approach the direction of strong radiation and enter the strong field area, where the mutual coupling increases. Therefore, our proposed stair-step distribution for the antenna array is able to not only significantly avoid scanning grating lobes but also efficiently decreases mutual coupling.

**Figure 11:**
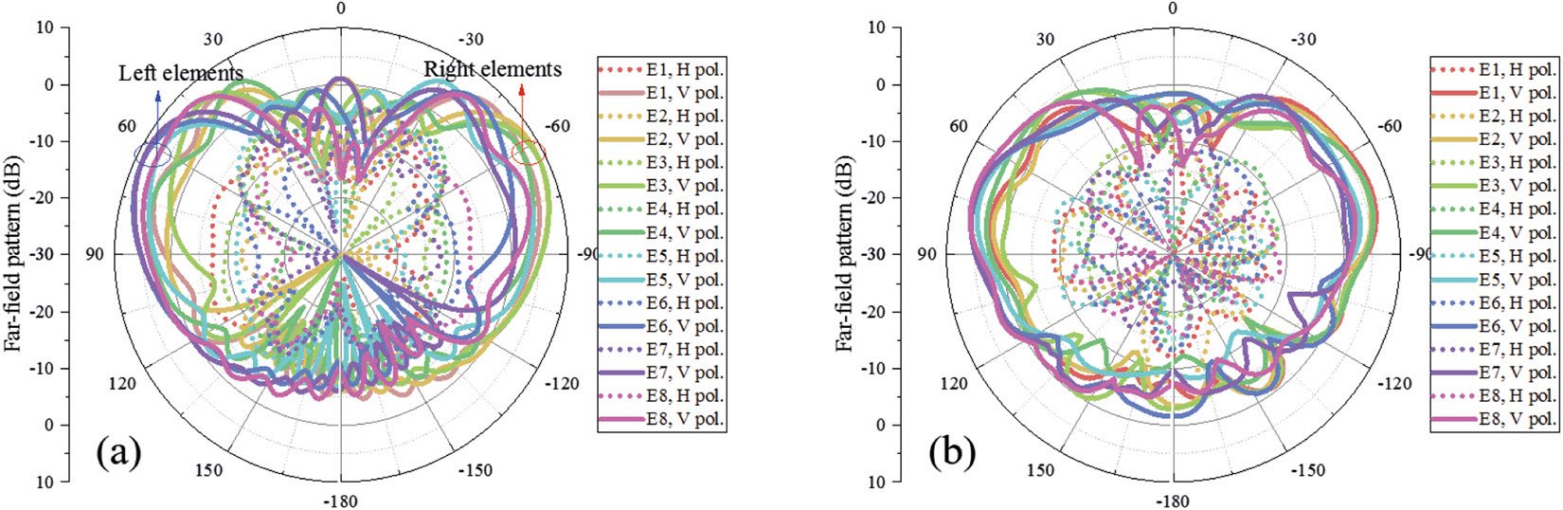
Active far-field radiation patterns of each element in the array. (**a**) Simulated and (**b**) measured active far-field radiation patterns in the yz plane. E1~E8 represent Element 1~Element 9, respectively. The H pol. and V pol. represent the horizontally polarized component and vertically polarized component, respectively. All the elements have bidirectional radiation patterns in both simulated and measured results shown in the figures, and the vertically polarized component is 15 dB stronger than the horizontally polarized component.

The active far-field radiation patterns of each element in the proposed array are shown in Fig. 11. All the elements have bidirectional radiation patterns in both the simulated and measured cases shown in the figures, and the vertically polarized component is 15 dB stronger than the horizontally polarized component. For each element, the two near-end-fire beams are asymmetrical in both the simulated and measured results because of the effect of the other elements in the array.

Subsequently, the time reversal synthesis method was used to adaptively determine the feeding amplitude and phase corresponding to the desired radiation pattern. Due to its ability to automatically focus, the time reversal technique has been widely used in super-resolution imaging^60–62^, high-capacity communications^63–66^, and phased array synthesis^47,67^. The time reversal synthesis method consists of the following characteristics^47,67^: (1) The excitation amplitude and phase of each element can be adaptively determined regardless of array arrangement and mutual coupling. (2) The excitation signals of all the elements can be determined by a time reversal operation both in simulations and in practical communication systems; therefore, time reversal is a solution for intelligent communication systems. It should be noted that the radiation efficiency of the antenna array can be reduced by the mutual coupling, although the main radiation beam of the array can be adaptively pointed to the desired directions using the time-reversal approach in situations of strong mutual coupling. Therefore, the strategies for reducing mutual coupling discussed in the previous paragraphs remain necessary. The basic process of the time reversal synthesis method is described as follows: In Step 1, the phased array is illuminated by detecting signals with temporal waveforms that cover the operating frequency bandwidth of the array from the desired steering directions. In Step 2, each element of the array receives the signal at the same time, and the time-reversal operation is applied. In Step 3, each element of the array transmits the time-reversed signal. In the frequency domain, the time reversal operation is equivalent to phase conjugation. Thus, the process can be finished in the frequency domain. The amplitude ${{\rm{A}}}_{n}$ and phase ${{\rm{\phi }}}_{n}$ at operational frequency ${f}_{0}$ of the received signal of *Element n* can be obtained by Fourier transform and the amplitude and phase of the corresponding exciting signal in Step 3 can be determined as ${{\rm{A}}}_{n}$ and $-{{\rm{\phi }}}_{n}$. It is difficult to control amplitude excitations in practical applications. It has been proven that the amplitude ${{\rm{A}}}_{n}$ of each element can be set uniformly to 1 in the time reversal synthesis process, with a weak effect on the radiation pattern of the array^47^. Therefore, only the phase is controlled using the time reversal synthesis method in this paper. In other words, in Step 3, each element of the array transmits a signal with an amplitude of 1 and a phase of $-{{\rm{\phi }}}_{n}$. In Step 4, the signal is focused in the directions of the detecting signals, so that the desired main beams will be obtained. The process of the time reversal synthesis method is shown in supplementary Movie 1.

**Figure 12:**
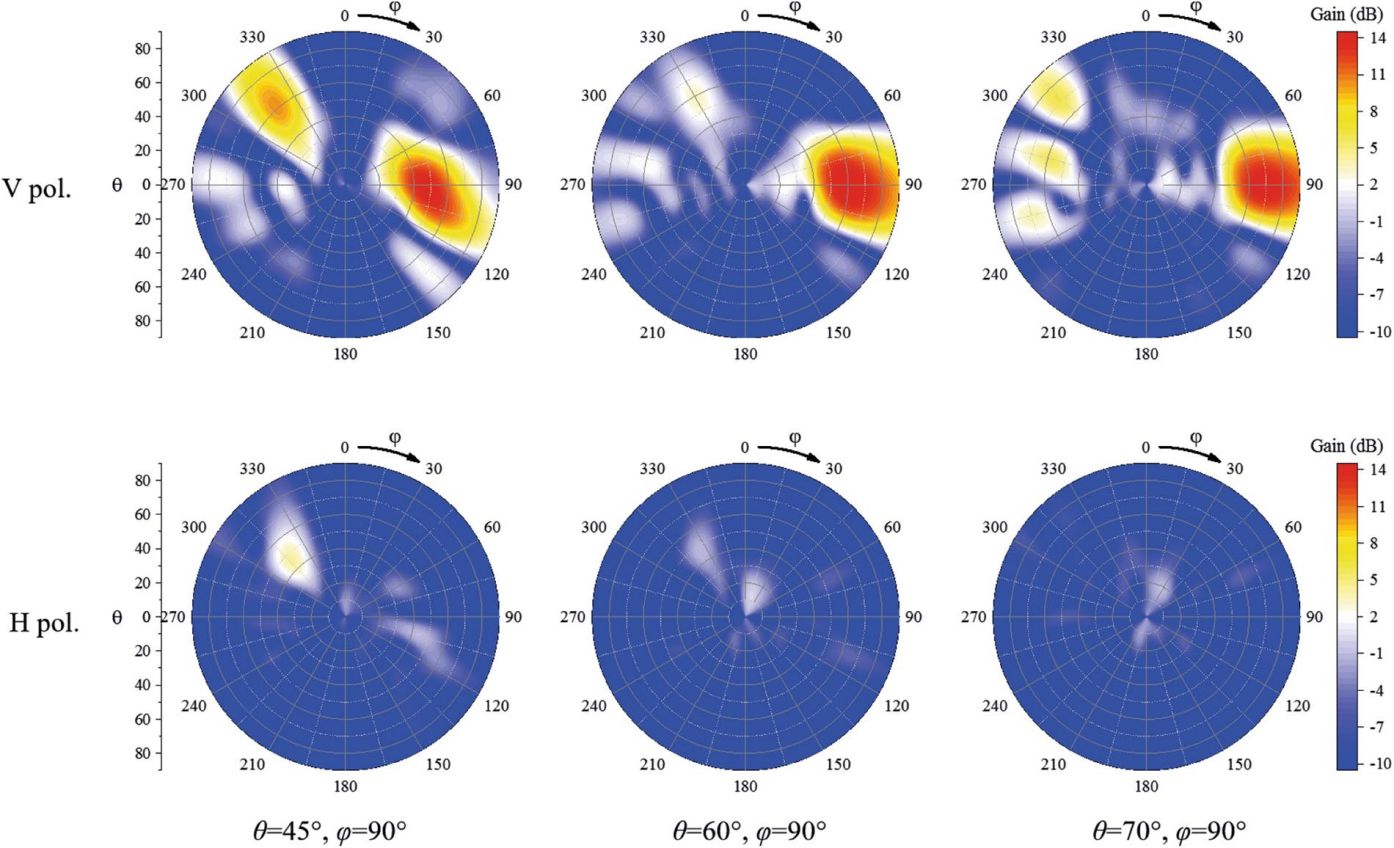
Simulated patterns of the array scanning in θ directions at 5.8 GHz. The H pol. and V pol. represent the horizontally polarized component and vertically polarized component, respectively.

**Figure 13:**
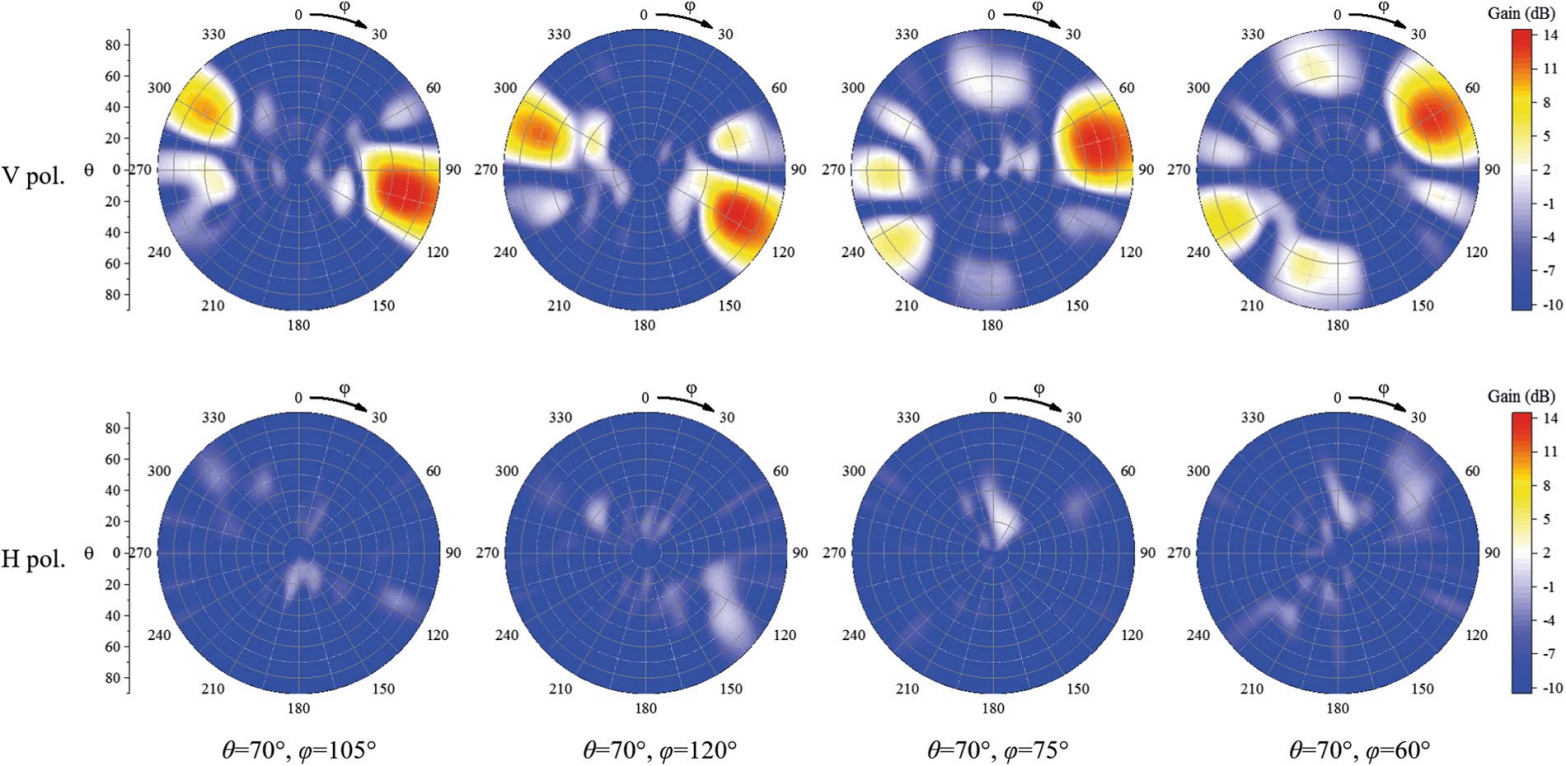
Simulated patterns of the array scanning in φ directions at 5.8 GHz. The H pol. and V pol. represent the horizontally polarized component and vertically polarized component, respectively.

Using the time reversal synthesis method, radiation patterns of the proposed array were synthesised. The simulated scanning patterns of the proposed array are shown in Figs 12 and 13. Given the structural symmetry of the proposed array, the scanning patterns around only one end-fire direction are shown in the figures; the scanning performances around the other end-fire direction are similar. From Fig. 12, we can see that when the main beam is directed to ${\rm{\theta }}=45^\circ ,{\rm{\phi }}=90^\circ$, the 3-dB beam in the yz plane approximately covers ${\rm{\theta }}=35^\circ$ to $\,{\rm{\theta }}=60^\circ$. When the main beam is directed to ${\rm{\theta }}=60^\circ ,{\rm{\phi }}=90^\circ$, the 3-dB beam in the yz plane approximately covers ${\rm{\theta }}=45^\circ$ to $\,{\rm{\theta }}=80^\circ$. When the main beam is directed to ${\rm{\theta }}=70^\circ ,{\rm{\phi }}=90^\circ$, the 3-dB beam in the yz plane approximately covers ${\rm{\theta }}=55^\circ$ to $\,{\rm{\theta }}=90^\circ$. From Fig. 13, we observe that the main beam of the array can be scanned from ${\rm{\theta }}=60^\circ$ to ${\rm{\phi }}=120^\circ$ and that the radiation beams of the proposed array can be scanned in the end-fire area. In addition, the horizontally polarized components in all the scanning cases are more than 10 dB smaller than the vertically polarized components.

**Figure 14:**
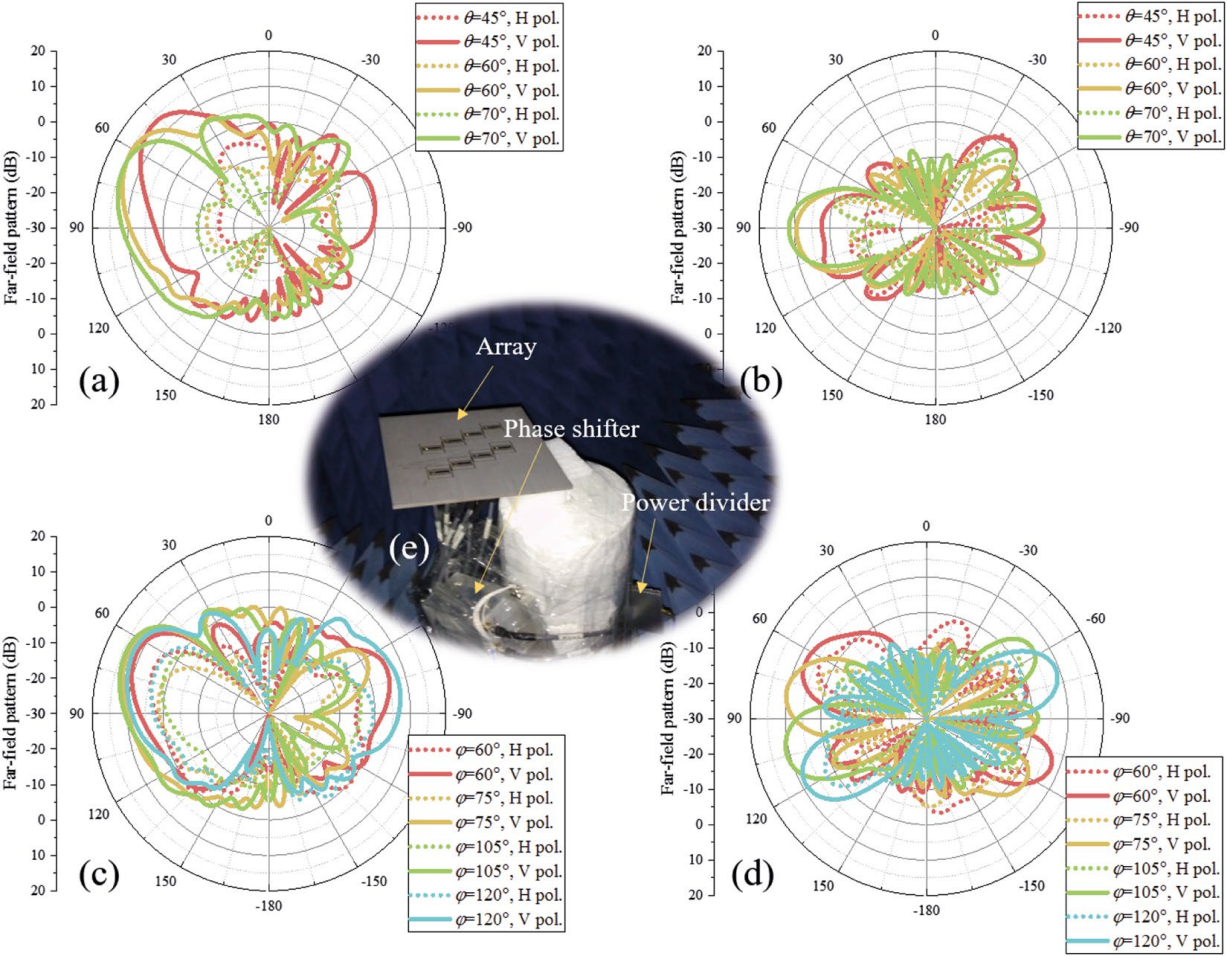
Measured scanning patterns of the array. (**a**) and (**b**) are patterns scanning in θ directions at 5.8 GHz in the yz plane (E plane) and xy plane, respectively. (**c**) and (**d**) are patterns scanning in φ directions at 5.8 GHz in the E planes and xy plane, respectively. E planes in φ = 60°, φ = 75°, φ = 105°, φ = 120° cases are φ = 60°/−120° plane, φ = 75°/−105° plane, φ = 105°/−75° plane, φ = 120°/−60° plane, respectively. The H pol. and V pol. represent the horizontally polarized component and vertically polarized component, respectively. (**e**) Photograph of pattern-measurement configuration. Ports of the measured array are connected to an 8-channel phase shifter, which is fed by source through a 1-to-8 power divider. Each channel of the phase shifter can be independently controlled by computer. In the measurement, the feed amplitude and phase of *Element n* in each case is set to 1 and $-{{\rm{\phi }}}_{n}$ based on the simulated results.

**Figure 15:**
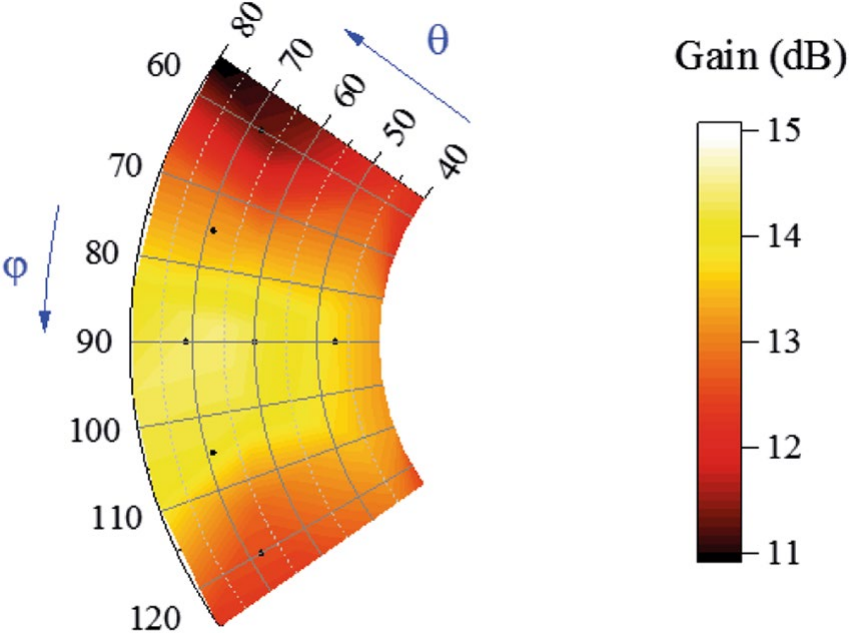
Measured scanning gains of the array at 5.8 GHz. Scanning gains in the near-end-fire directions (60° < φ < 120°, 45° < θ < 80°) are within 11~15 dB.

The measured scanning radiation patterns are shown in Fig. 14 around only one end-fire direction of the proposed array as a result of the symmetry of the structure. When the main beam is directed to ${\rm{\theta }}=45^\circ ,{\rm{\phi }}=90^\circ$, the 3-dB radiation beam in the yz plane approximately covers ${\rm{\theta }}=36^\circ$ to $\,{\rm{\theta }}=65^\circ$. When the main beam is directed to ${\rm{\theta }}=60^\circ ,{\rm{\phi }}=90^\circ$, the 3-dB beam in the yz plane approximately covers ${\rm{\theta }}=44^\circ$ to $\,{\rm{\theta }}=84^\circ$. When the main beam is directed to ${\rm{\theta }}=70^\circ ,{\rm{\phi }}=90^\circ$, the 3-dB beam in the yz plane approximately covers ${\rm{\theta }}=50^\circ$ to $\,{\rm{\theta }}=90^\circ$. When the radiation beam is scanned in the yz plane, the horizontally polarized components in all the scanning cases are approximately 15 dB smaller than the vertically polarized components. When the beam is scanned in the xy plane, the main beam of the array can be scanned from ${\rm{\phi }}=60^\circ$ to ${\rm{\phi }}=120^\circ$ and the horizontally polarized components in all the scanning cases are more than 6 dB smaller than the vertically polarized components. The measured scanning gains of the proposed array are shown in Fig. 15. From Fig. 15, we can see that the scanning gain decreases when the main beam direction is away from the end-fire direction. The scanning gains in the near-end-fire directions (60° < φ < 120°, 45° < θ < 80°) are within 11~15 dB, and the gain fluctuations are small, which indicates good scanning performance.

**Figure 16:**
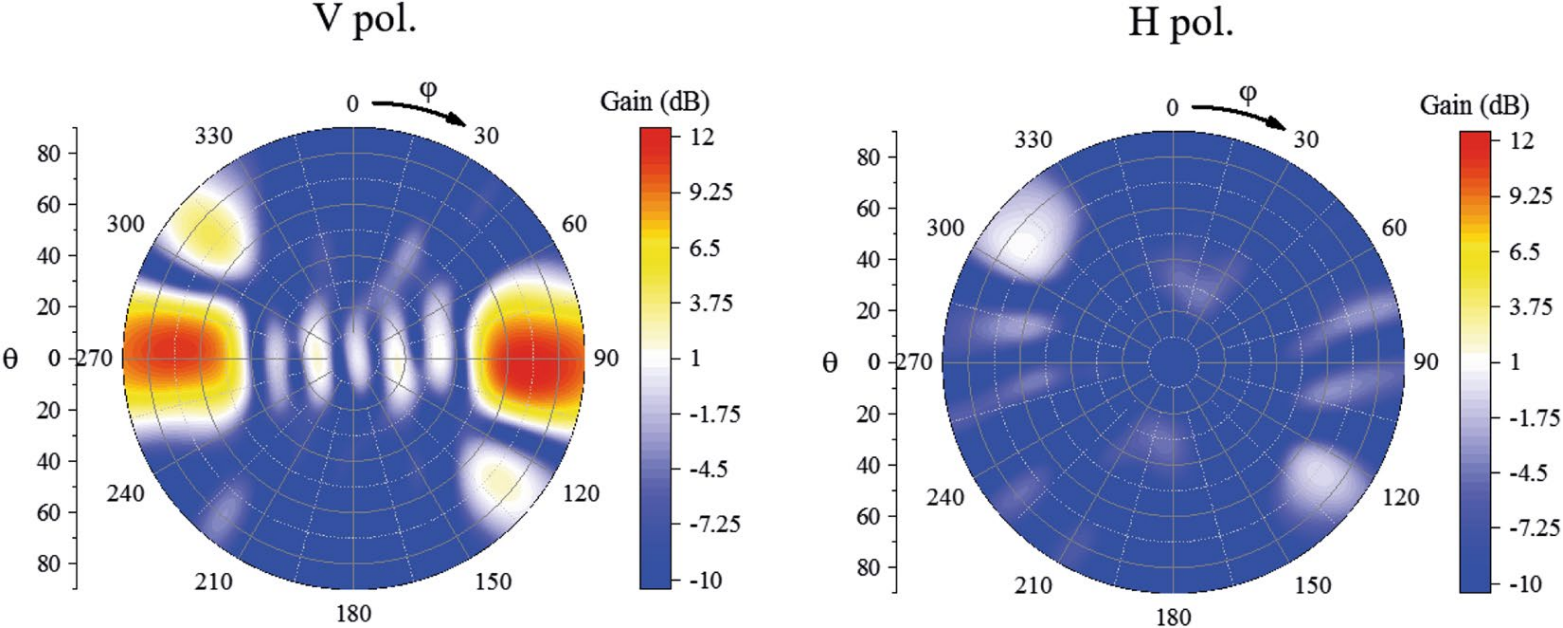
Bidirectional radiation patterns of the array. The H pol. and V pol. represent the horizontally polarized component and vertically polarized component, respectively. The 3-dB beamwidth of the vertically polarized component can cover bidirectional near-end-fire directions, including ${\rm{\theta }}=90^\circ ,{\rm{\phi }}=90^\circ$ and $\,{\rm{\theta }}=90^\circ ,{\rm{\phi }}=270^\circ$.

The above results show the scanning performances in the near-end-fire directions of the proposed array, which indicate that the proposed array can scan its main beam in hotspot areas of tunnel environments. In a time reversal communication system, after a user transmits a detection signal in the tunnel, the proposed array can automatically focus its main beam to the user using a tracking mechanism. In this situation, one main radiation beam can increase the transmission efficiency and avoid signal mutual interference. In other situations, for example, when users are located in two opposite directions and transmit detection signals simultaneously, the proposed array is expected to have multi-beam performance. To demonstrate the multi-beam performance, the proposed array is simultaneously illuminated by two plane waves from ±y, i.e., ${\rm{\theta }}=90^\circ ,{\rm{\phi }}=90^\circ$ and $\,{\rm{\theta }}=90^\circ ,{\rm{\phi }}=270^\circ$. Based on the time reversal synthesis method introduced above, a bidirectional end-fire radiation pattern can be realized, as shown in Fig. 16. The 3-dB beamwidth of the vertically polarized component can cover the bidirectional near-end-fire directions, including ${\rm{\theta }}=90^\circ ,{\rm{\phi }}=90^\circ$ and $\,{\rm{\theta }}=90^\circ ,{\rm{\phi }}=270^\circ$. The horizontally polarized component is 10 dB smaller than the vertically polarized component, and an excellent bidirectional radiation pattern is demonstrated. From the above analysis, we can see that the proposed array can be used in both single-user and multi-user situations in tunnel environments.

## Discussion

In this study of a planar phased array for use in tunnel environments, we implemented three key techniques for the proposed array: the bidirectional element design method, the bidirectional array arrangement method, and the adaptive array synthesis method. To design the bidirectional element, the primary concept was to manipulate dual-magnetic currents based on a phase-inversed binary array. Moreover, the dual-magnetic-current antenna was realized using a patch with opposite short edges and opposite open edges. To increase the operational frequency bandwidth, two slots were etched on the patch of the antenna to excite two bidirectional modes. In addition, the two contradictory issues of reducing strong coupling and avoiding grating lobes were solved using the decoupling cavity and the sparse array configuration in the proposed tunnel-used array arrangement process. Finally, the time reversal synthesis method was applied to synthesize array patterns. The time reversal method can be used not only to synthesize desired radiation patterns in simulations but also to manipulate the focus of radiation beams in practical intelligent communication systems. With the time reversal method, the proposed array can adaptively generate a single beam or multiple beams according to the positions of users in tunnel environments. In conclusion, excellent beam-scanning performances can be realized using the proposed array and methods. The proposed methods can be used to improve bandwidth and reduce energy consumption in demanding wireless communication applications in tunnel environments.

## Methods

### Simulation method

The proposed bidirectional antenna and planar phased array were simulated and optimized using CST Microwave Studio. The selected operating frequency band of the proposed antenna and array was centred around 5.8 GHz, which is a part of the spectrum open for academic research in China. The time reversal synthesis method was implemented in CST Microwave Studio using the following steps: (1) The proposed array is illuminated by a plane wave of 0–10 GHz from the desired steering directions. (2) Each element of the array receives the signal at the same time and the received signals are exported. (3) The amplitude ${{\rm{A}}}_{n}$ and phase ${{\rm{\phi }}}_{n}$ at 5.8 GHz of the received signal of Element **n** are calculated by Fourier transform in Matlab. (4) Array Element n transmits a signal with an amplitude of 1 and a phase of $-{{\boldsymbol{\phi }}}_{{\boldsymbol{n}}}$, and the desired far-field radiation pattern is obtained. When multiple beams are required, the illumination process should be performed several times because only one plane wave can be set during a calculation cycle in CST Microwave Studio. For example, two illumination calculations were performed in CST Microwave Studio to obtain Fig. 16. First, the proposed array was illuminated by a plane wave from the y direction, and the received signal at each port was exported. Subsequently, the proposed array was illuminated by a plane wave from the -y direction, and the received signal at each port was exported. Finally, the signals received in the two calculation steps at each port were added, and the Fourier transform of the added signals was calculated in Matlab to obtain the exciting phase for each port.

### Measurement method

The scattering matrices *S*_*mn*_ (*m*, *n* = 1, 2, 3, …, 8) for the proposed planar phased array were measured using an E8361A Vector Network Analyzer. When the S matrices of the two ports were measured, the other ports were connected to matched loads. The far-field radiation patterns of the proposed antenna and array were measured in a microwave anechoic chamber equipped with a SATIMO measurement system. When the active patterns of one element were measured, the other ports were connected to matched loads. The scanning performances were measured using the feed system shown in Fig. 14(e). Eight ports of the measured array were connected to an eight-channel phase shifter, which was fed by a source through a one-to-eight power divider. The phase shifter was controlled by computer software, and each phase channel could be controlled independently. In the measurement, the feed amplitude and phase of Element *n* in each case were set to 1 and $-{{\boldsymbol{\phi }}}_{{\boldsymbol{n}}}$, respectively, based on the results simulated in CST Microwave Studio and the Matlab calculations (see the section on the simulation method).

### Data availability

The data from this paper can be obtained from the University of Southampton ePrints research repository: http://doi.org/10.5258/SOTON/D0292.

## Electronic supplementary material

Movie 1
