# Publisher Correction: Planar array with bidirectional elements for tunnel environments

**DOI:** 10.1038/s41598-018-27403-3

**Published:** 2018-06-13

**Authors:** Ren Wang, Bing-Zhong Wang, Xiao Ding, Jun-Yu Ou

**Affiliations:** 1 Institute of Applied Physics, University of Electronic Science and Technology of China, Chengdu, 610054 China; 2 Optoelectronics Research Centre and Centre for Photonic Metamaterials, University of Southampton, Highfield, Southampton, SO17 1BJ UK

Correction to: *Scientific Reports* 10.1038/s41598-017-15817-4, published online 13 November 2017

The original version of this Article contained a repeated typographical error in the ‘Design Procedures and Results’ section under the subheading ‘Array with bidirectional elements for use in tunnels’ where,

‘*ϕnϕn’*

now reads:

‘*ϕn’*

This error has now been corrected in the HTML version of the Article; the PDF version was correct from the time of publication.

