# Supplementary figures and images for: Planar array with bidirectional elements for tunnel environments
Source: Sci Rep. 2017 Nov 13;7:15421. doi: 10.1038/s41598-017-15817-4 (PMC5684329; doi:10.1038/s41598-017-15817-4)

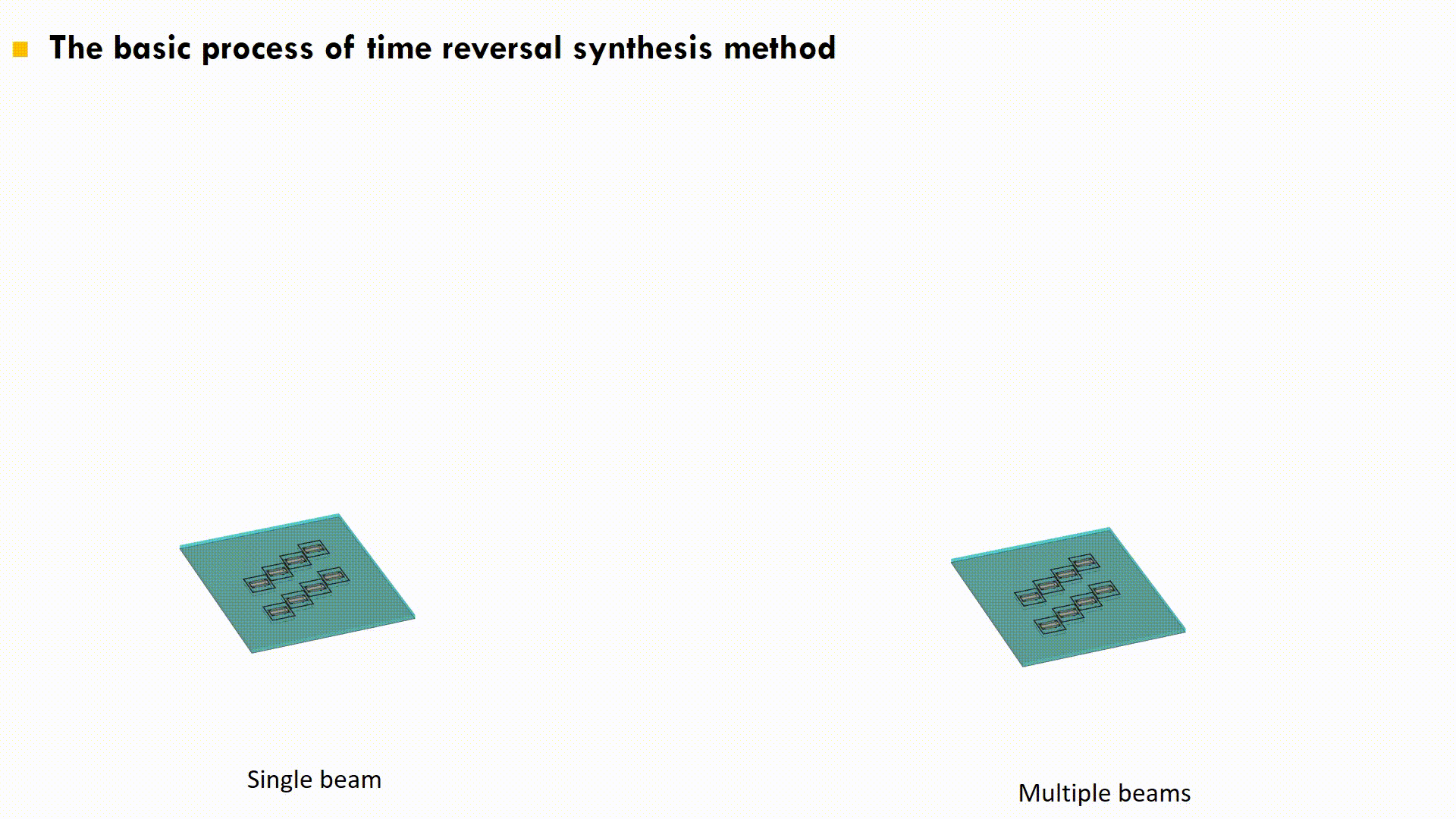

Supplement: Supplementary file 1 — Movie 1 [file 41598_2017_15817_MOESM1_ESM.gif]
